# Combined Analysis of the Aberrant Epigenetic Alteration of Pancreatic Ductal Adenocarcinoma

**DOI:** 10.1155/2019/9379864

**Published:** 2019-12-28

**Authors:** Rui Xu, Qiuyan Xu, Guanglei Huang, Xinhai Yin, Jianguo Zhu, Yikun Peng, Jukun Song

**Affiliations:** ^1^Department of Radiology, Guizhou Provincial People's Hospital, Guiyang, Guizhou, China; ^2^Department of Oral and Maxillofacial Surgery, Guizhou Provincial People's Hospital, Guiyang, Guizhou, China; ^3^Department of Urology, Guizhou Provincial People's Hospital, Guiyang, Guizhou, China; ^4^Department of Otorhinolaryngology-Head and Neck Surgery, Guizhou Provincial People's Hospital, Guiyang, Guizhou, China

## Abstract

**Background:**

Pancreatic ductal adenocarcinoma (PDAC) remains one of the most fatal malignancies due to its high morbidity and mortality. DNA methylation exerts a vital part in the development of PDAC. However, a mechanistic role of mutual interactions between DNA methylation and mRNA as epigenetic regulators on transcriptomic alterations and its correlation with clinical outcomes such as survival have remained largely uncovered in cancer. Therefore, elucidation of aberrant epigenetic alteration in the development of PDAC is an urgent problem to be solved. In this work, we conduct an integrative epigenetic analysis of PDAC to identify aberrant DNA methylation-driven cancer genes during the occurrence of cancer.

**Methods:**

DNA methylation matrix and mRNA profile were obtained from the TCGA database. The integration of methylation and gene expression datasets was analyzed using an R package MethylMix. The genes with hypomethylation/hypermethylation were further validated in the Kaplan–Meier analysis. The correlation analysis of gene expression and aberrant DNA methylation was also conducted. We performed a pathway analysis on aberrant DNG methylation genes identified by MethylMix criteria using ConsensusPathDB.

**Results:**

188 patients with both methylation data and mRNA data were considered eligible. A mixture model was constructed, and differential methylation genes in normal and tumor groups using the Wilcoxon rank test was performed. With the inclusion criteria, 95 differential methylation genes were detected. Among these genes, 74 hypermethylation and 21 hypomethylation genes were found. The pathway analysis revealed an increase in hypermethylation of genes involved in ATP-sensitive potassium channels, Robo4, and VEGF signaling pathways crosstalk, and generic transcription pathway.

**Conclusion:**

Integrated analysis of the aberrant epigenetic alteration in pancreatic ductal adenocarcinoma indicated that differentially methylated genes could play a vital role in the occurrence of PDAC by bioinformatics analysis. The present work can help clinicians to elaborate on the function of differentially methylated expressed genes and pathways in PDAC. CDO1, GJD2, ID4, NOL4, PAX6, TRIM58, and ZNF382 might act as aberrantly DNA-methylated biomarkers for early screening and therapy of PDAC in the future.

## 1. Introduction

Pancreatic ductal adenocarcinoma (PDAC) is still one of the primary health problems due to high mortality and incidence worldwide. PDAC remains the primary cause of cancer-related mortality worldwide. It is reported that a 5-year survival rate remains lower, and the average survival time is no more than six months [1]. PDAC is the fourth primary cause of cancer death affecting 56,670 new patients in 2017 in the USA [2, 3]. Although the advances in surgical techniques and chemoradiotherapy protocols had largely improved, the overall survival of PDAC patients remains poor. Meanwhile, due to resistant to radiotherapy and chemotherapy in patients with PDAC, little progress has been made related to its therapy in the past decades [4]. Therefore, to reduce mortality and improve the treatment of PDAC, we need to find new early diagnostic biomarkers and therapeutic targets for early detection and risk classification of PDAC.

DNA methylation has previously been found to be a valuable biomarker for several cancers [5–7]. The epigenetic variations usually suppress protein translation and gene transcription in human carcinogenesis. Several studies have demonstrated that DNA methylation exerted an early event, and new efforts are focused on finding biomarkers for early disease detection, prognostication, and treatment selection, especially in multiple cancers [8–11]. Therefore, elaborating the potential mechanisms during the initiation and development of cancer would greatly improve the diagnosis, treatment, and prognosis evaluation. Abnormal methylation could affect the functions of crucial genes by altering their expression. In this study, we utilized systemic analysis to identify a group of novel gene signatures, which may be regulated by DNA methylation. In addition, the present study can help clinicians to elaborate on the function of DMGs in PDAC. Our study might be the groundwork for further elucidation of the PDAC mechanism and screening of the diagnostic biomarkers for the early stage of PDAC.

## 2. Materials and Methods

### 2.1. Data Source and Data Processing

In the current study, the mRNA expression and DNA methylation data of the PDAC cohort were obtained from the TCGA data portal (https://tcga-data.nci.nih.gov/tcga/, August 28, 2018). The 4 adjacent nontumor pancreatic tissues and 187 PDAC samples were included in the gene expression profiles, where the mRNA microarray employed IlluminaHiSeq RNA-Seq array, while 10 adjacent nontumor control tissues and 178 PDAC tissues were included in the gene methylation dataset, where the methylation microarray used Illumina HumanMethylation 450 BeadChip.

The DEGList and calcNormFacors functions in the edgeR package were employed to normalize the RNA sequence data and DNA methylation data [12]. Both tumor samples and normal samples were used in the same way.

### 2.2. Integrative Analysis

Through the integration of gene expression and DNA methylation datasets, the MethylMix package in R software was employed to recognize DNA methylation-driven cancer genes [13]. There are three steps to detect DNA methylation-driven cancer genes between the DNA methylation and gene expression datasets. First, the correlation between gene methylation and gene expression level was imputed, and significant correlation genes were found. Second, a beta mixture model was constructed to determine a methylation state across multiple patients. Third, the Wilcoxon rank sum test was employed to compare DNA methylation states between tumor and normal samples. A cutoff of 0.05 was considered statistically significant. The hypomethylation genes were defined as positive differential methylation (DM), while hypermethylation genes were regarded as negative DM.

### 2.3. Survival Analysis

To further explore the correlation of DNA hypermethylation or hypomethylation genes with overall analysis, the Kaplan–Meier survival analysis and univariate Cox regression analysis were conducted to analyze DNA methylation genes. The log-rank test was employed to compare the survival difference between the PDAC and nontumor samples. A two-sided *P* value of <0.05 was defined as statistically significant. The R “Survival” package was used to identify independent prognostic variables.

### 2.4. Pathway Analysis

The pathway analysis was analyzed by the ConsensusPathDB website (http://cpdb.molgen.mpg.de/), which integrated interaction networks in *Homo sapiens* including protein-protein, gene regulatory, genetic, signaling, metabolic, and drug-target interactions, as well as biochemical pathways [14]. The pathway analysis was performed using the prognostic DNA methylation-driven gene lists produced by MethylMix. The pathway analysis was conducted on the hypermethylation genes and hypomethylation genes, respectively.

## 3. Results

### 3.1. Demography

After excluding those patients with a survival of less than one month, 178 patients were included in the study. The clinical and pathological information of the cohort study is exhibited in Table 1. In the whole cohort, 1.12% of patients were less than 35–39 years old, 10.11% were 40–49 years old, 20.79% were 50–59 years old, 29.78% were 60–69 years old, 29.21% were 70–79 years old, and 8.99% were above 80 years old. The median follow-up duration was 46.0 months (range, 2–119 months). There were, respectively, 19 PDCA patients with pathologic TNM stage I, 147 patients with pathologic TNM stage II, 4 patients with pathologic TNM stage III, 5 patients with pathologic TNM stage IV, and 3 patients with an unknown TNM stage in our study. By the end of the last follow-up, 94 (52.81%) patients of the entire population had died.

### 3.2. Identifying Methylation-Driven Cancer Genes

**Figure 1:**
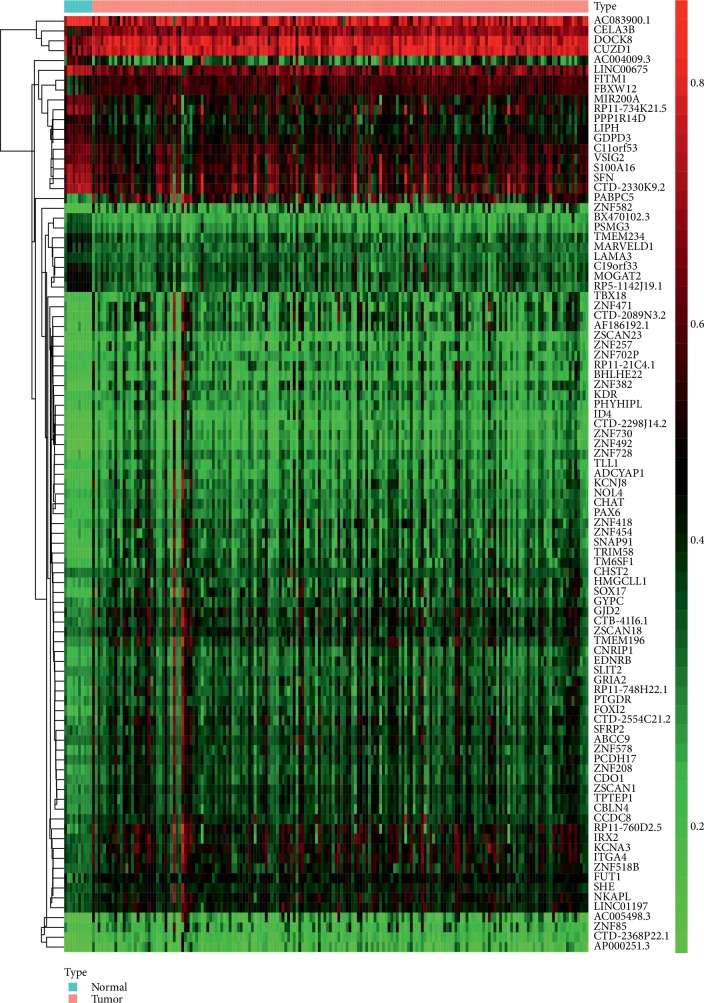
Representative heat map of the 74 differential methylation genes. Red represents upregulation; blue represents downregulation.

A combined approach was utilized to assess the epigenetic alterations that may be involved in the occurrence of the PDAC. The DNA methylation-driven cancer genes were screened using the MethylMix package in R software. The 95 genes were recognized as differential DNA methylation genes when adjusted *P* value <0.05 and cor*P* value <−0.3 were set as the threshold for differential methylation genes (DMGs). Among these genes, 74 genes (77.89%) were hypermethylation genes, and the remainder of genes were hypomethylation genes (Supplementary [Supplementary-material supplementary-material-1]). The heat map is shown in Figure 1.

### 3.3. Correlation Analysis between DNA Methylation Genes and mRNA

**Figure 2:**
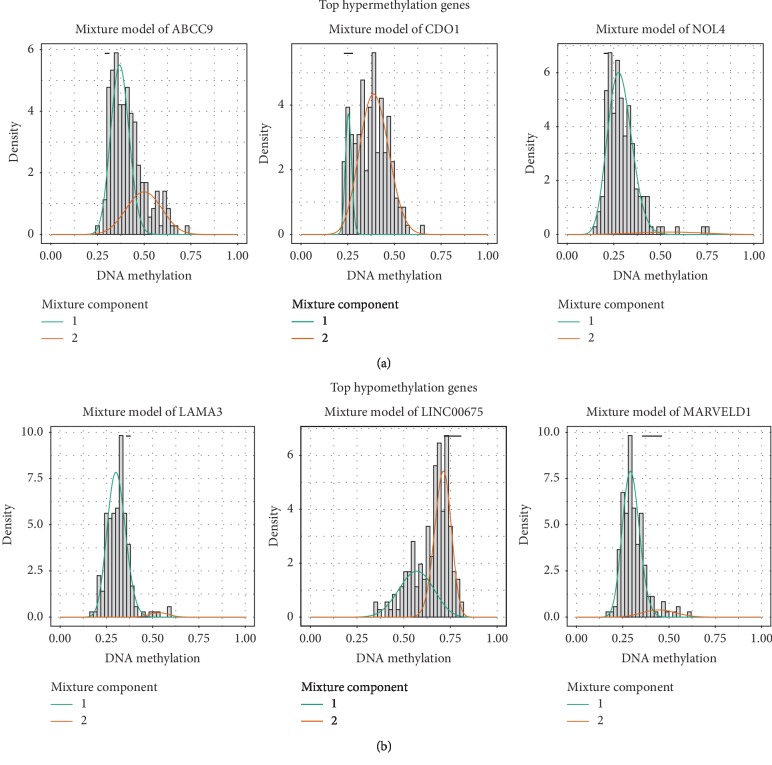
Summary of (a) top three hypermethylated and (b) top three hypomethylated genes. The abscissa is the degree of methylation, the ordinate is the number of methylated samples, the histogram represents the methylation distribution of the tumor samples, and the curve demonstrates the simulated trend curve of the methylation distribution in the tumor samples. The black horizontal line above the graph is the methylation level distribution of the normal samples. The red line represents the distribution of methylation in tumor samples.

**Figure 3:**
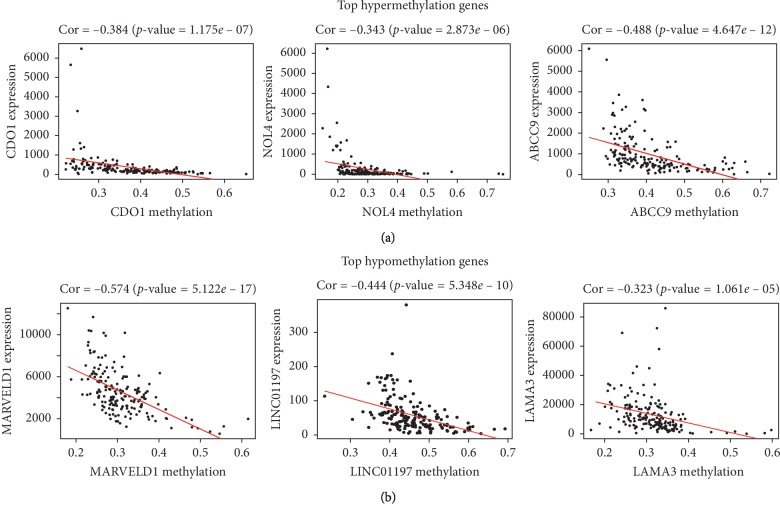
Correlation analysis between gene expression and hypermethylated/hypomethylated genes.

Among 95 differential methylation genes, 74 genes exhibited higher methylation levels in tumor samples compared with normal samples and were referred to as hypermethylation genes, while 21 genes were defined as hypomethylation genes. The top five hypermethylated/hypomethylated genes are shown in Figure 2. All methylation-driven cancer genes showed a negative association between DNA methylation genes and mRNA. The top five hypermethylated/hypomethylated genes are also exhibited in Figure 3.

### 3.4. Survival Analysis

**Figure 4:**
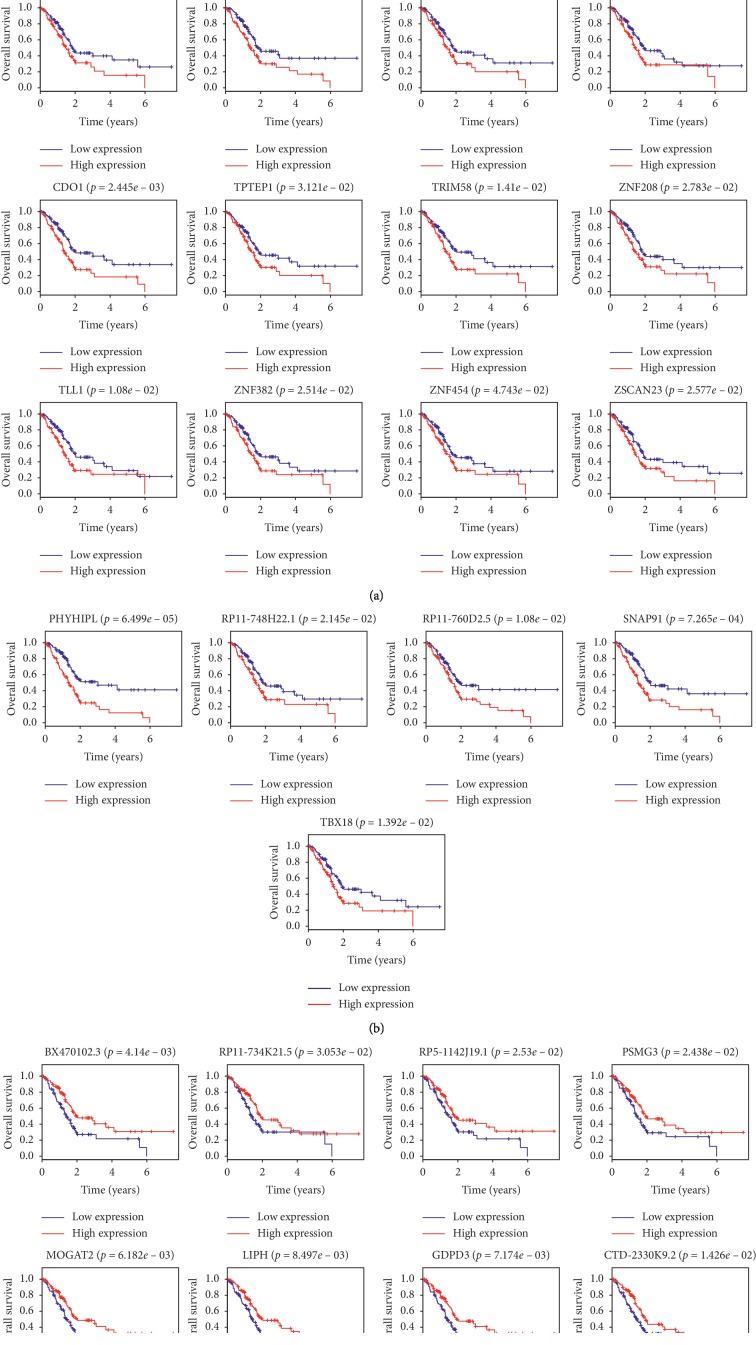
Kaplan–Meier survival curves for overall survival outcomes according to the risk cutoff point for prognostic hypermethylated/hypomethylated genes. The *P* value of the log-rank test is less than 0.01.

In order to evaluate the effect of differential genes on PDAC patient's prognosis, we conducted the Kaplan–Meier survival analysis and univariate Cox regression analysis. The findings indicated that 25 out of 74 hypermethylation genes and 10 out of 21 hypomethylation were associated with the patient's overall analysis (Table 2). Patients with higher expression in the hypermethylation group exhibited poorer OS than those who have lower expression. However, patients with lower expression in the hypomethylation group demonstrated poorer OS than those who have lower expression. Kaplan–Meier curves for the high-risk and low-risk groups are observed in Figure 4.

### 3.5. Pathway Analysis

**Figure 5:**
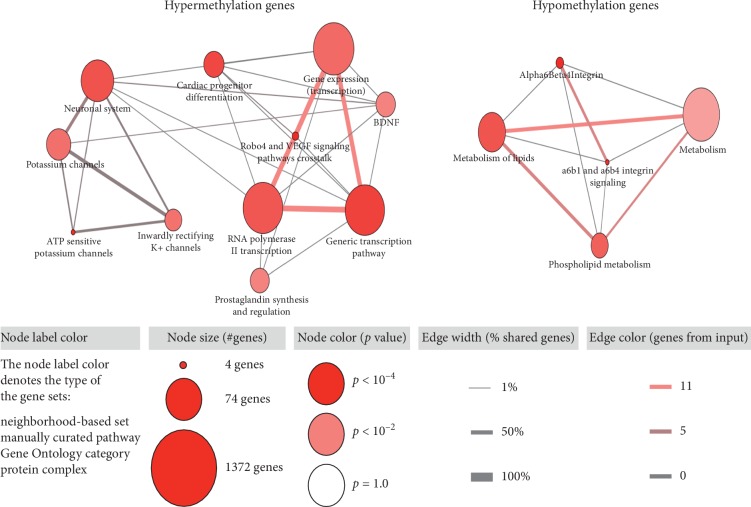
The pathways enriched for hypermethylated and hypomethylated genes in the TCGA PDAC cohort.

To explore the potential functional implication of DNA methylation-driven cancer genes, we performed the pathway analysis by ConsensusPathDB. Several pathways are identified in Figure 5. For hypermethylated genes, pathways were mainly enriched in Robo4 and VEGF signaling pathways crosstalk, ATP-sensitive potassium channels, and generic transcription pathway. For hypomethylated genes, a total of 4 pathways focusing on the biological pathways were enriched, including a6b1 and a6b4 Integrin signaling, metabolism of lipids, and phospholipid metabolism reactome.

## 4. Discussion

The PDCA is characterized by late diagnosis, poor prognosis, low rates of overall survival, and locoregional recurrences. The primary validated treatment selection remains surgical resection. Local recurrence is a primary cause of failure to treatment [15]. Despite several factors were identified biomarkers for early detection and develop new treatments in PDAC, the overall survival rate and prognosis remain poor [16, 17]. Meanwhile, due to an absence of particular symptoms at an early stage, along with resistance to therapies, high metastatic ability, and lack of diagnostic biomarkers and screening methods, early diagnosis remains the primary treatment option in PDAC. Therefore, it was urgent to explore the potential mechanisms and pathogenesis during the development and progression of PDCA and to uncover new biomarkers and therapeutic targets.

Epigenetic altercation exerts a vital part in carcinogenesis and tumor development progression. Aberrant methylation could affect the functions of crucial genes by altering their expression. Several studies have demonstrated that DNA methylation is referred to as an early phenomenon, and new efforts are focused on recognizing biomarkers of early disease detection, prognostication, and treatment option selection, especially in PDCA [5–7, 18, 19]. DNA hypomethylation has also been documented to be involved in the occurrence of tumors and alters genome rearrangement and chromosomal instability [20, 21]. Therefore, elaborating on the potential mechanisms of development of PDCA would largely elevate the diagnosis and improve the treatment and prognosis evaluation.

In current works, we integrated DNA methylation data and mRNA data and screen DNA methylation-driven cancer genes, and Kaplan–Meier survival analysis was further validated these prognostic results. Compared to normal groups, 95 differential methylation genes (74 hypermethylation genes and 21 hypomethylation gens) were found in the tumor group. We also found that patients with hypermethylation yielded poor-prognosis modifications, demonstrating that many combinations of hypermethylation modifications contribute to poor prognosis. The pathway analysis was also performed, and the results indicated that Robo4 and VEGF signaling pathways crosstalk and ATP-sensitive potassium channels may be related to the development and progression of PDAC. One important result from the pathway analysis was involved in the vascular endothelial growth factor (VEGF) pathway among hypermethylated genes. It is widely accepted that VEGF is a vital driver of the angiogenic modification in physiological and pathological processes in both embryo and adult. VEGF is often found overexpressed in tumors [22]. VEGF exerts a crucial role in vascular homeostasis and the maintenance of vascular integrity. The VEGF signal transduction pathway has identified as an important therapeutic target for patients with many cancers [23, 24]. The two hypermethylated genes (SLIT2 and KDR) were enriched in the pathway. The methylation of SLIT2 was associated with the development and progression of hepatocellular carcinoma [25], dysplasia of pancreatic cystic neoplasms [26], breast cancer [27], and nasopharyngeal carcinoma [28]. The methylation of KDR was also correlated with the development and progression of oral squamous cell carcinoma [29].

Several prognostic hypermethylated genes had been shown to be correlated with a variety of cancers in prior studies (Table 3). A growing body of evidence indicated that CDO1 promoter methylation was correlated with many cancers. Kojima et al. suggested that the hypermethylated gene of CDO1 served as biomarkers and contributed to colorectal cancer [30]. Brait et al. reported that CDO1 serves as a tumor suppressor and is deactivated by promoter methylation in several tumors [31]. Jeschke et al. demonstrated that the silence of CDO1 may account for the survival of breast cancer cells and resistance to anthracyclines [32]. Yang et al. reported the methylation status of the CDO1 promoter to become a diagnostic biomarker for hepatitis B virus-related HCC [33]. CDO1 promoter methylation was also associated with the risk of gastric cancer [34], breast cancer [35], hepatocellular carcinoma [36], and prostate cancer [37]. Sirnes et al. reported that GJC1 promoter methylation played a crucial role in colorectal cancer [38] and follicular lymphoma [39]. ID4 serves as hypermethylation gene and tumor suppressor gene in breast cancer [40, 41] and acute leukemia [42, 43]. ID4 promoter methylation was also correlated with the risk of prostate cancer [44, 45]. Meanwhile, NOL4, PAX6, TRIM58, and ZNF382 promoter methylation was also associated with the occurrence of many cancers [46–55].

Integrated analysis of the aberrant epigenetic alteration in PDAC indicated that differentially methylated genes may be involved in the occurrence of PDAC. Moreover, the present study can help clinicians to elaborate on the function of differentially methylated expressed genes in PDAC. Our study might be the groundwork for further mechanisms elucidation of PDAC and identification of the diagnostic biomarkers for an early stage of PDAC.

## Figures and Tables

**Table 1:** Clinical characteristics.

Clinical variables	Clinical values (*N* = 185)
Sex (male/female)	98/80
Age (mean ± std)	64.70 ± 11.13
Race (Asian/black/white/NA)	11/7/155/14
Pathologica stage (I/II/III/IV/V/NA)	19/21/147/4/5/3
Pathologic_T stage (T1/T2/T3/T4/NA)	7/21/144/4/2
Pathologic_N stage (N0/N1/NA)	48/125/5
Pathologic_N stage (M0/M1/NA)	83/5/90
Grade (G1/G2/G3/G4/G*x*/NA)	29/94/50/2/3

**Table 2:** Prognostic hypermethylation/hypomethylation genes for PDAC in Kaplan–Meier survival analysis and univariate Cox regression analysis.

Gene	*P* value (KM)	HR	Low 95%	High 95%	*P* value (Cox)
ID4	0.042672	3.630678	0.520149	25.34241	0.193383
CBLN4	0.023498	8.850718	1.048889	74.68398	0.045088
NOL4	0.004024	4.335809	0.684543	27.46246	0.119342
ZSCAN23	0.025771	2.565136	0.291866	22.54433	0.395617
ZNF208	0.027825	4.378881	0.610815	31.39183	0.14171
TPTEP1	0.031211	9.031061	0.565839	144.1401	0.119457
CTD-2554C21.2	0.02653	3.226591	0.659874	15.77709	0.148008
HMGCLL1	0.032094	5.418468	0.99398	29.53761	0.050821
TBX18	0.013916	1.719498	0.399601	7.399053	0.466623
CDO1	0.002445	9.153049	1.051298	79.69033	0.044934
GJD2	0.03223	2.621186	0.475785	14.44059	0.268377
KCNJ8	0.047027	2.946227	0.607266	14.29399	0.179935
ZNF382	0.025139	2.887076	0.625426	13.32725	0.174279
RP11-748H22.1	0.021448	2.767153	0.603237	12.69342	0.190326
AC005498.3	0.037114	3.33757	0.887535	12.55091	0.074517
KCNA3	0.017149	1.742724	0.2681	11.3282	0.560838
TLL1	0.010797	5.624124	0.745679	42.41878	0.093876
ZNF454	0.047434	2.430637	0.414374	14.25765	0.325139
GRIA2	0.007244	4.082081	0.778325	21.40929	0.096198
SNAP91	0.000726	4.182435	0.957687	18.26563	0.057108
PHYHIPL	6.50E-05	4.99462	0.648914	38.44306	0.122433
PAX6	0.045649	3.477117	0.489556	24.69657	0.212806
TRIM58	0.014099	2.686691	0.452754	15.9431	0.276686
RP11-760D2.5	0.010799	3.659025	0.898893	14.89439	0.070119
PABPC5	0.029366	2.600122	0.768849	8.79319	0.124263
PSMG3	0.024383	0.070082	0.005646	0.869985	0.038608
BX470102.3	0.00414	0.056038	0.003752	0.836902	0.036704
CTD-2330K9.2	0.014257	0.234518	0.064259	0.855889	0.028124
RP11-734K21.5	0.030528	0.143102	0.033485	0.611557	0.008702
GDPD3	0.007174	0.056417	0.005122	0.621427	0.018844
C19orf33	0.042501	0.239202	0.03509	1.630583	0.144101
MOGAT2	0.006182	0.040403	0.00416	0.392444	0.005669
RP5-1142J19.1	0.025301	0.111422	0.015353	0.808636	0.030006
C11orf53	0.013738	0.265261	0.031938	2.20313	0.219203
LIPH	0.008497	0.087046	0.010817	0.700467	0.021758

**Table 3:** Literature search of key hypermethylation genes screened by MethylMix criteria.

Gene symbol	Gene name	Chromosome	Tumor suppressor gene in cancer	Hypermethylated gene in cancer	Altered pathways	Cancer type
CDO1	Cysteine dioxygenase type 1	5q22.3		Hypermethylation	Viral mRNA translation; metabolism	Colorectal cancer; hepatocellular carcinoma; gastric cancer; prostate cancer; esophageal squamous cell carcinoma
GJD2	Gap junction protein delta 2	15q14		Hypermethylation	Gap junction; G-beta gamma signaling	Colorectal cancer
ID4	Inhibitor of DNA binding 4, HLH protein	6p22.3	Tumor suppressor gene	Hypermethylation	TGF-beta signaling pathway (KEGG); signaling pathways regulating pluripotency of stem cells	Breast cancer; acute leukemia
NOL4	Nucleolar protein 4	18q12.1	Tumor suppressor gene			Head and neck cancer; cervical cancer
PAX6	Paired box 6	11p13		Hypermethylation		Gastric cancer; breast cancer
TRIM58	Tripartite motif containing 58	1q44		Hypermethylation		Colorectal cancer; lung squamous cell carcinoma; hepatocellular carcinoma
ZNF382	Zinc finger protein 382	19q13.12	Tumor suppressor gene	Hypermethylation	Generic transcription pathway	Gastric cancer; pediatric acute myeloid leukemia

## Data Availability

The data used to support the findings of this study could be obtained from the TCGA website.

## Supplementary Materials

Supplementary Materials Supplementary Table 1: identifying methylation-driven cancer genes in PDAC.
