# Supplementary material for: Combined Analysis of the Aberrant Epigenetic Alteration of Pancreatic Ductal Adenocarcinoma
Source: Biomed Res Int. 2019 Dec 28;2019:9379864. doi: 10.1155/2019/9379864 (PMC6949667; doi:10.1155/2019/9379864)
Supplement: Supplementary Materials — Supplementary Table 1: identifying methylation-driven cancer genes in PDAC. [file 9379864.f1.pdf]

Supplementary Table 1: Identifying methylation driven cancer genes in PDAC.

gene	normalMean	TumorMean	logFC	pValue	adjustP	cor	corPavlue
ADCYAP1	0.112865	0.268029	1.247792	4.43E-07	0.000171	-0.34712	2.06E-06
CHAT	0.179846	0.305369	0.763791	6.94E-07	0.000268	-0.31901	1.42E-05
TRIM58	0.12391	0.32373	1.385493	1.02E-06	0.000392	-0.4638	7.03E-11
ABCC9	0.302053	0.412768	0.45053	1.26E-06	0.000485	-0.48841	4.65E-12
SOX17	0.169685	0.377733	1.154511	2.41E-06	0.000932	-0.43018	2.06E-09
ZNF257	0.091935	0.236028	1.360268	2.56E-06	0.000988	-0.52099	8.97E-14
ZNF382	0.077315	0.28135	1.863547	2.96E-06	0.001143	-0.55356	1.11E-15
ZNF730	0.06397	0.224437	1.810845	3.09E-06	0.001194	-0.40968	1.36E-08
KCNJ8	0.155232	0.291815	0.910634	3.63E-06	0.001401	-0.41293	1.02E-08
TPTEP1	0.269209	0.384339	0.513652	4.07E-06	0.001571	-0.38441	1.17E-07
FOXI2	0.184259	0.373064	1.017687	4.07E-06	0.001571	-0.33473	4.95E-06
NKAPL	0.342089	0.460865	0.429971	4.83E-06	0.001866	-0.4	3.17E-08
ZSCAN1	0.274006	0.40699	0.570787	4.90E-06	0.001893	-0.36757	4.48E-07
SHE	0.343212	0.429225	0.322632	4.97E-06	0.00192	-0.48088	1.09E-11
LINC01197	0.347562	0.456013	0.391804	5.26E-06	0.002032	-0.44405	5.35E-10
CTD-2368P22.1	0.057404	0.159913	1.478056	5.42E-06	0.002091	-0.38132	1.51E-07
ZSCAN23	0.102916	0.202051	0.973251	5.73E-06	0.002212	-0.5081	4.49E-13
TMEM196	0.257568	0.408826	0.666533	5.90E-06	0.002276	-0.33145	6.20E-06
ZNF492	0.061905	0.211811	1.774659	6.24E-06	0.002408	-0.50853	4.27E-13
GJD2	0.27374	0.433708	0.663918	6.42E-06	0.002477	-0.35143	1.51E-06
KDR	0.163614	0.260843	0.672885	6.60E-06	0.002548	-0.39763	3.88E-08
KCNA3	0.320998	0.483295	0.590341	6.98E-06	0.002695	-0.50703	5.13E-13
BX470102.3	0.32381	0.203769	-0.66821	7.38E-06	0.00285	-0.44025	7.78E-10
PSMG3	0.350581	0.237299	-0.56304	7.59E-06	0.002931	-0.45861	1.21E-10
ZNF728	0.105199	0.269407	1.356664	7.59E-06	0.002931	-0.34545	2.33E-06
ZNF518B	0.310312	0.444683	0.519055	7.81E-06	0.003014	-0.65476	3.65E-23
CTD-2089N3.2	0.120879	0.312336	1.369535	8.03E-06	0.0031	-0.36058	7.65E-07

ZNF578	0.23309	0.380361	0.706481	8.73E-06	0.00337	-0.41414	9.11E-09
IRX2	0.327397	0.458801	0.48683	9.23E-06	0.003562	-0.45585	1.62E-10
CTD-2298J14.2	0.08524	0.205151	1.267094	9.75E-06	0.003765	-0.40271	2.51E-08
ZNF208	0.229628	0.370305	0.689415	1.00E-05	0.00387	-0.5121	2.75E-13
SNAP91	0.15278	0.317379	1.054756	1.03E-05	0.003979	-0.36339	6.18E-07
RP11-21C4.1	0.15228	0.283519	0.896715	1.12E-05	0.004321	-0.3674	4.54E-07
ZNF454	0.161489	0.319138	0.982745	1.15E-05	0.004441	-0.65384	4.41E-23
AF186192.1	0.130348	0.313501	1.266098	1.18E-05	0.004565	-0.45606	1.58E-10
MOGAT2	0.462807	0.348343	-0.4099	1.51E-05	0.005834	-0.35091	1.57E-06
PABPC5	0.260792	0.493929	0.921405	1.55E-05	0.005994	-0.50261	8.76E-13
CNRIP1	0.204353	0.335082	0.713447	1.64E-05	0.006327	-0.43367	1.48E-09
ZSCAN18	0.308259	0.419568	0.44476	1.64E-05	0.006327	-0.56888	1.19E-16
FITM1	0.444177	0.575615	0.373971	1.68E-05	0.0065	-0.55081	1.64E-15
DOCK8	0.672113	0.804547	0.259473	1.68E-05	0.0065	-0.50951	3.78E-13
PCDH17	0.287294	0.388388	0.434969	1.73E-05	0.006678	-0.44268	6.13E-10
CELA3B	0.532732	0.701517	0.397068	1.78E-05	0.00686	-0.59381	2.42E-18
ID4	0.087056	0.200525	1.203774	1.78E-05	0.00686	-0.52716	4.05E-14
ZNF418	0.201325	0.341689	0.763159	1.83E-05	0.007047	-0.70179	1.02E-27
S100A16	0.71221	0.566845	-0.32935	1.88E-05	0.007239	-0.58711	7.12E-18
PTGDR	0.246552	0.361059	0.550347	1.93E-05	0.007436	-0.32526	9.42E-06
EDNRB	0.214877	0.343524	0.676903	1.93E-05	0.007436	-0.45724	1.40E-10
ZNF702P	0.164447	0.243464	0.566084	1.98E-05	0.007637	-0.45387	1.98E-10
GYPC	0.251714	0.359443	0.513975	1.98E-05	0.007637	-0.59834	1.15E-18
NOL4	0.208533	0.293877	0.494935	1.98E-05	0.007638	-0.3425	2.87E-06
SFN	0.726957	0.543613	-0.41929	2.09E-05	0.008057	-0.54358	4.49E-15
RP5-1142J19.1	0.460692	0.310145	-0.57086	2.32E-05	0.008962	-0.40129	2.84E-08
GRIA2	0.232955	0.363083	0.640248	2.38E-05	0.009203	-0.39723	4.02E-08
CDO1	0.25361	0.372026	0.552789	2.42E-05	0.009326	-0.3844	1.17E-07
CBLN4	0.257805	0.383099	0.571435	2.45E-05	0.009451	-0.37188	3.20E-07
TMEM234	0.429244	0.31844	-0.43078	2.94E-05	0.011366	-0.36435	5.74E-07
MIR200A	0.633701	0.492496	-0.36369	3.02E-05	0.011668	-0.32502	9.57E-06
RP11-760D2.5	0.291483	0.471652	0.694314	3.02E-05	0.011669	-0.44159	6.83E-10
ZNF471	0.116317	0.297197	1.353357	3.27E-05	0.012621	-0.50641	5.52E-13
C11orf53	0.652834	0.536969	-0.28188	3.44E-05	0.013297	-0.51156	2.94E-13
CTB-41I6.1	0.28347	0.4116	0.538047	3.54E-05	0.013648	-0.42919	2.26E-09
RP11-748H22.1	0.220534	0.367601	0.737143	3.68E-05	0.01419	-0.40353	2.34E-08
TM6SF1	0.196485	0.341937	0.799313	4.13E-05	0.015943	-0.32377	1.04E-05
ZNF85	0.12224	0.216208	0.822704	4.24E-05	0.01636	-0.53749	1.03E-14
CUZD1	0.668302	0.812044	0.281058	4.46E-05	0.017224	-0.69523	4.96E-27
HMGCLL1	0.214986	0.353255	0.716471	4.46E-05	0.017224	-0.40395	2.25E-08
PPP1R14D	0.547627	0.444988	-0.29943	4.58E-05	0.017672	-0.47341	2.50E-11
SLIT2	0.262294	0.340397	0.376034	4.70E-05	0.018131	-0.39791	3.79E-08
LIPH	0.565237	0.455834	-0.31035	4.70E-05	0.018131	-0.55576	8.13E-16
AP000251.3	0.045693	0.144137	1.657406	5.07E-05	0.019578	-0.36094	7.44E-07

GDPD3	0.596104	0.490846	-0.28029	5.34E-05	0.020602	-0.36524	5.36E-07
CTD-2330K9.2	0.751258	0.563838	-0.41403	5.34E-05	0.020602	-0.37473	2.56E-07
AC004009.3	0.530847	0.334725	-0.66532	5.76E-05	0.022235	-0.47271	2.69E-11
BHLHE22	0.127556	0.219774	0.784894	5.91E-05	0.022805	-0.3332	5.50E-06
LAMA3	0.368665	0.313096	-0.23571	5.91E-05	0.022805	-0.32347	1.06E-05
TBX18	0.141154	0.2758	0.966355	5.98E-05	0.023096	-0.39238	6.06E-08
PHYHIPL	0.143839	0.244314	0.76428	6.06E-05	0.02339	-0.34618	2.21E-06
CTD-2554C21.2	0.258129	0.406858	0.65643	7.23E-05	0.027898	-0.55153	1.48E-15
LINC00675	0.761384	0.651446	-0.22498	7.60E-05	0.02933	-0.61417	7.74E-20
FUT1	0.341635	0.458052	0.423058	7.69E-05	0.029698	-0.44229	6.37E-10
AC083900.1	0.88682	0.779764	-0.1856	7.79E-05	0.030071	-0.32395	1.03E-05
FBXW12	0.444273	0.572056	0.364709	7.79E-05	0.030071	-0.65438	3.95E-23
ITGA4	0.332801	0.462757	0.475595	7.79E-05	0.030072	-0.46986	3.67E-11
CCDC8	0.321361	0.419157	0.383293	8.39E-05	0.032405	-0.50923	3.91E-13
PAX6	0.180263	0.291337	0.692588	8.82E-05	0.034054	-0.36111	7.34E-07
AC005498.3	0.061349	0.212491	1.792288	9.16E-05	0.035342	-0.34268	2.84E-06
RP11-734K21.5	0.671682	0.502106	-0.41979	9.27E-05	0.035782	-0.38518	1.10E-07
C19orf33	0.453114	0.333937	-0.4403	9.27E-05	0.035782	-0.56643	1.71E-16
CHST2	0.293002	0.368273	0.329866	9.50E-05	0.036677	-0.32536	9.36E-06
ZNF582	0.102169	0.233333	1.191432	9.74E-05	0.037593	-0.52485	5.46E-14
SFRP2	0.284453	0.386057	0.440623	0.000121	0.046867	-0.33111	6.35E-06
MARVELD1	0.40787	0.30897	-0.40064	0.000124	0.04802	-0.57445	5.12E-17
VSIG2	0.638975	0.52554	-0.28196	0.000127	0.049202	-0.64681	1.79E-22
TLL1	0.1738	0.252424	0.53842	0.000127	0.049202	-0.37648	2.23E-07
